# Multigene panel predicting survival of patients with colon cancer

**DOI:** 10.7150/jca.35902

**Published:** 2019-11-01

**Authors:** Hao-Dong Li, Chen Liang

**Affiliations:** 1 President's Office, Zhongnan hospital of Wuhan University, Hubei province, China, 430071; 2 Office of quality and safety management, Zhongnan hospital of Wuhan University, Hubei province, China, 430071

**Keywords:** colon cancer, elastic net, Cox proportional hazards regression model

## Abstract

**Objective:** The purpose of this study was to investigate multigene panel markers to predict long-term survival in patients with colon cancer.

**Methods and materials:** GSE39582 was randomly divided into a training set and a validation set, while TCGA-COAD and GSE17536 were treated as two independent validation cohorts. Survival-associated genes were included in elastic net penalized Cox proportional hazards regression (ENCPH) model. Based on the results of the ENCPH, a multigene panel was constructed. We evaluated predictive performance of the multigene panel by univariate and multivariate survival analysis, and time-dependent ROC analysis.

**Results:** A total of 1025 colon cancer patients were included in the study, and 94 genes were showed to be related with the overall survival of colon cancer patients, of which 7 genes were integrated to construct a multigene panel according to ENCPH model. The multigene panel could stratify colon cancer patients into notably different risk groups in the training set and three verification cohort. Results of multivariable CPH model suggested that the multigene panel was an independent prognostication factor. The multigene-containing nomogram showed reliable prediction ability on the 3- and 5-year survival of colon cancer patients with internally and externally validated C-indexes exceeded 0.7.

**Conclusion:** The multigene panel we introduced showed considerable prognosis performance in colon cancer, and the multigene panel containing nomogram would help clinicians assess long-term survival probability.

## Introduction

Colon cancer is one of the common malignant tumors that seriously endanger human health 1. Due to the ageing of the population, lifestyle changes, and advances in diagnostic techniques, approximately 1.4 million new cases of colon cancer are diagnosed and 690,000 colon cancer related deaths are recorded each year 2. Surgery, cryosurgery, radiation therapy, chemotherapy, and targeted therapy are well established management for colon cancer 3, 4. Pathological stage is widely accepted to the key determinant of the prognosis and treatment of patients with colon cancer 3, 5. Although surgery can treat nearly 50% of early stage colon cancer, the vast majority will relapse and often lead to death 5, 6. Postoperative managements are widely recommended for patients with advanced stage colon cancer^7^. Chemotherapy, which often uses different drugs or drug combinations to inhibit the proliferation of tumor cells, is often used after surgical treatment and inevitably injures normal cells while killing tumor cells owning to its non-target effect 5, 8-10. Therefore, in addition to well established pathological stage, identification of novel biomarkers related with the genetics heterogeneity of colon cancer might help the prognostication stratification and treatment individualization.

Existing colon cancer gene expression studies offer the possibility of the identification of novel biomarkers 11-14. Thus, in the present study, we use an elastic network algorithm to integrate existing colon cancer gene expression study to find new colon cancer markers associated with the recurrence and prognosis of colon cancer patients.

## Methods and Materials

### Colon cancer mRNA expression studies

Colon cancer gene expression study GSE39582 11, measured by Affymetrix Human Genome U133 Plus 2.0 Array, consisted of 585 colon cancer samples. We obtained the RMA normalized mRNA expression data and the corresponding clinical data (including age, gender, TNM stage, tumor location, overall survival, recurrence free survival) of the associated colon cancer patients from the Gene Expression Omnibus (GEO) (https://www.ncbi.nlm.nih.gov/geo/). We treated the GSE39582 cohort as a discovery set, and randomly (in a 1:1 ratio) categorized colon patients in this cohort into a training set and test set. The mRNA expression profile of TCGA colon cancer cohort (TCGA-COAD)^14^ consisted of 329 colon samples, colon cancer samples with clinical information (including age, gender, histological type, preoperative CEA, pathological TNM stage, recurrence free survival and overall survival) were included in the study. We obtained the levels 3 mRNA expression profile (log2(x+1) transformed RSEM normalized count) from the UCSC Xena (https://xenabrowser.net/datapages/). We treated the TCGA-COAD as an independent validation set in this study. GSE17536 12, 13, measured by Affymetrix Human Genome U133 Plus 2.0 Array, included 177 colon cancer samples. We obtained the RMA^15^ normalized mRNA expression data and the corresponding clinical information of patients with colon cancer (age, gender, race, AJCC-stage, grade, overall survival, disease free survival) of GSE17536 from the GEO database.

### Model construction

At first, we identified overall survival (OS) associated genes (genes at P value less than 0.0001) in the discovery set (GSE39582) using univariate Cox proportional hazards regression (CPH) model^16^. Then, the discovery set was divided into two subgroups as mentioned above. In the training set, elastic net regularized CPH (ENCPH) model was performed. To fit the optimal model, we performed 10-fold cross-validation to tune the two hyperparameters α and λ. After that, we built a multigene-based prognostication combination on the basis of the fitted ENCPH 17.

### Assessment of the prognostication performance of the multigene signature

Time-dependent receiver operating characteristic curve (ROC)^18^ at one-year, three-year, five-year, seven-year, ten-year, and fifteen-year was applied to assess the prognostication performance of the multigene panel in the training set, test set and validation set using the R package “survivalROC”. Univariate CPH model and multivariable CPH model were performed to assess the OS, recurrence-free survival (RFS) and disease-free survival (DFS) of colon cancer patients in the different risk groups derived from the cutoff value through time-dependent ROC analysis.

### Development and validation of a multigene containing nomogram

Nomogram, which included several lines corresponding to certain clinical parameters, was widely used to predict the survival probability of patients in clinical settings 19. Thus, we tried to construct the multigene containing nomogram by including the age, gender, TNM stage, tumor location, and the multigene panel. The nomogram was formed, validated with 1000 bootstrapping internally and externally, and calibrated at 3-year and 5-year using the R package “rms”. Decision curve analysis (DCA) analysis was conducted to assess the clinical application prospects of the Multigene panel in the training set 20.

### Comparison of the prognostication performance of our multigene panel with existing multigene markers

C-index, also known as concordance” statistic or C-statistic, is a measure of goodness of fit for survival outcomes in a CPH model, and higher C-index means higher predictive ability^21^. Therefore, to further confirm the performance of our multigene panel, we compared the C-indexes, calculated by using the R package “survcomp 22” , with a total of 10 biomarkers reported by others 23-33. Student T test was used to compare C-indexes between two groups.

### Gene set enrichment analysis (GSEA) to investigate the mechanisms related with the multigene panel

Finally, we performed GSEA^34^ to analyze the molecular bases that related with function of the multigene panel on the survival of colon cancer patients. “c5.bp.v6.2.symbols” and “c2.cp.kegg.v6.2.symbols” was used to perform Gene Ontology (GO) and Kyoto Encyclopedia of Genes and Genomes (KEGG) enrichment analysis, respectively. Colon samples in the training set were classified into significantly different risk groups based on the cutoff mention above. Any gene set enriched with P value less than 0.05 and false discovery rate less than 0.25 were regarded as significantly enriched.

## Results

### Demographic characteristic of colon patients

The training set included 287 patients with colon cancer, of which 134 were female and 153 were male, and the median age of these patients were 67.8 years (range: 22-97) (supplementary table s1). The test set included 288 patients with colon cancer, of which 122 were female and 164 were male, and the median age of these patients were 69 years (range: 24.9-96). The independent validation set TCGA-COAD included 275 patients with colon cancer, of which 124 were female and 151 were male, and the median age of these patients were 67 years (range: 31-90). The independent validation set GSE17536 included 177 patients with colon cancer, of which 81 were female and 96 were male, and the median age of these patients were 66 years (range: 26-92). More details regarding the characteristics of patients in the above four cohorts were shown in supplementary table s1-3.

### Development of 9-gene combination for predicting the survival of colon cancer patients

After univariate CPH analysis, a total of 92 genes were shown to significantly (P<0.0001) associated the overall survival patients in the GSE39582 cohort. We included the 92 survival associated genes into the ENCPH model fitted with the optimal hyperparameter (alpha=0.078, lambda=5.3734) calculated through 10-fold cross-validation (supplementary figure s1), According to the result of feature selection, MYB (MYB proto-oncogene, transcription factor), MSLN(mesothelin), INHBB (inhibin subunit beta B), DCBLD2 (discoidin, CUB and LCCL domain containing 2), MAP1B (microtubule-associated protein 1B), PRELID2 (PRELI domain containing 2), and SH3RF2 (SH3 domain containing ring finger 2) were finally used to build multigene panel for predicting the survival of colon cancer patients. The risk score of each colon cancer patients were estimated based on the coefficients and the expression levels of these genes (supplementary table s4). Then, patients in the four cohorts were categorized into significantly risk group based on the optimal cutoff value on the basis of the results of time-dependent ROC analysis (the cutoff values were 1, 0.999, 0.063 and -0.001 in the training set, test set, TCGA-COAD, and GSE17536, respectively).

### Prediction value of the multigene panel

**Figure 1:**
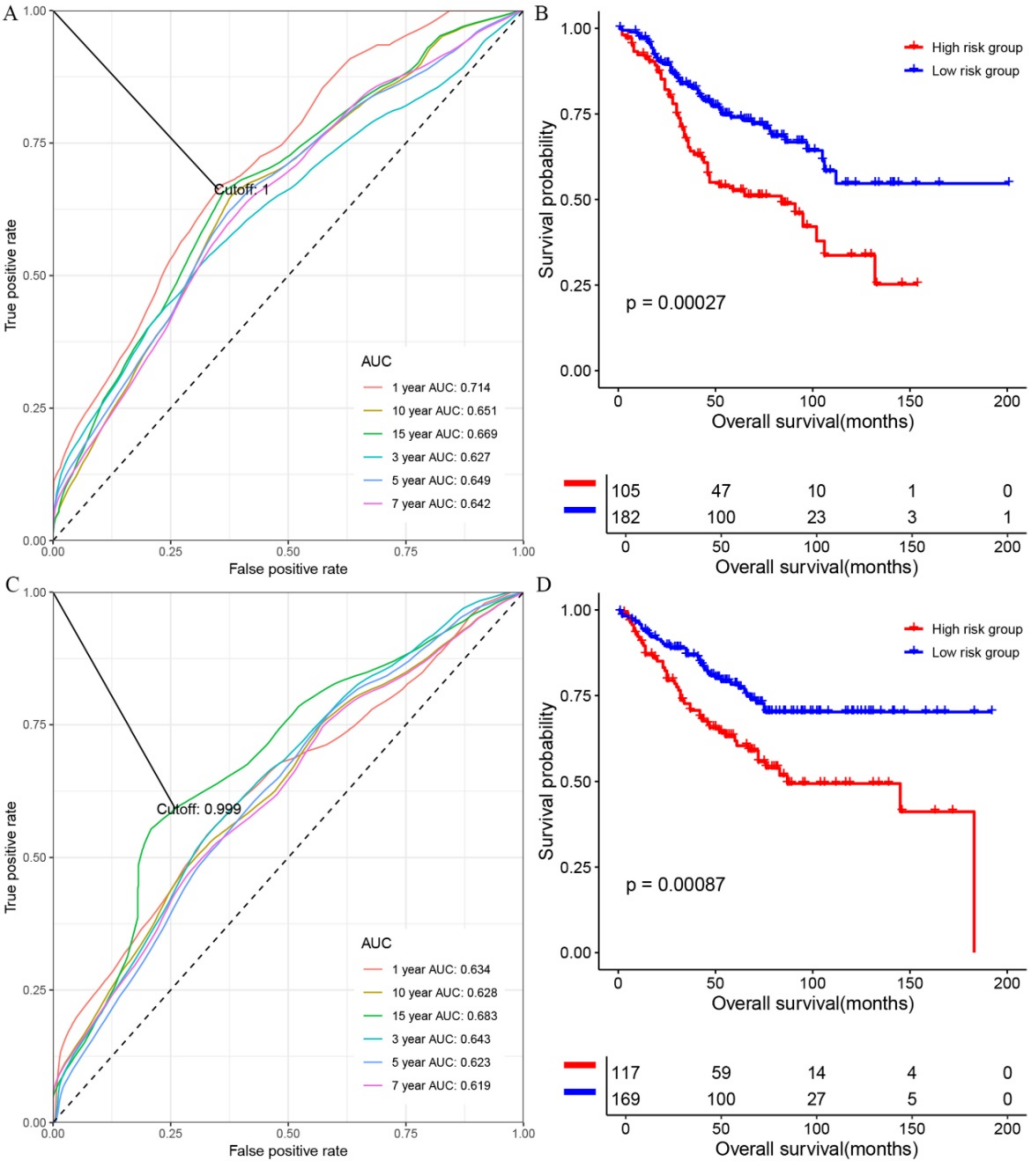
The performance of the multigene combination in predicting the overall survival patients with colon cancer in the training set and test set. (A) Time-dependent analysis in the training set. (B) Overall survival differences of patients in the training set. (C) Time-dependent analysis in the test set. (B) Overall survival differences of patients in the test set.

**Figure 2:**
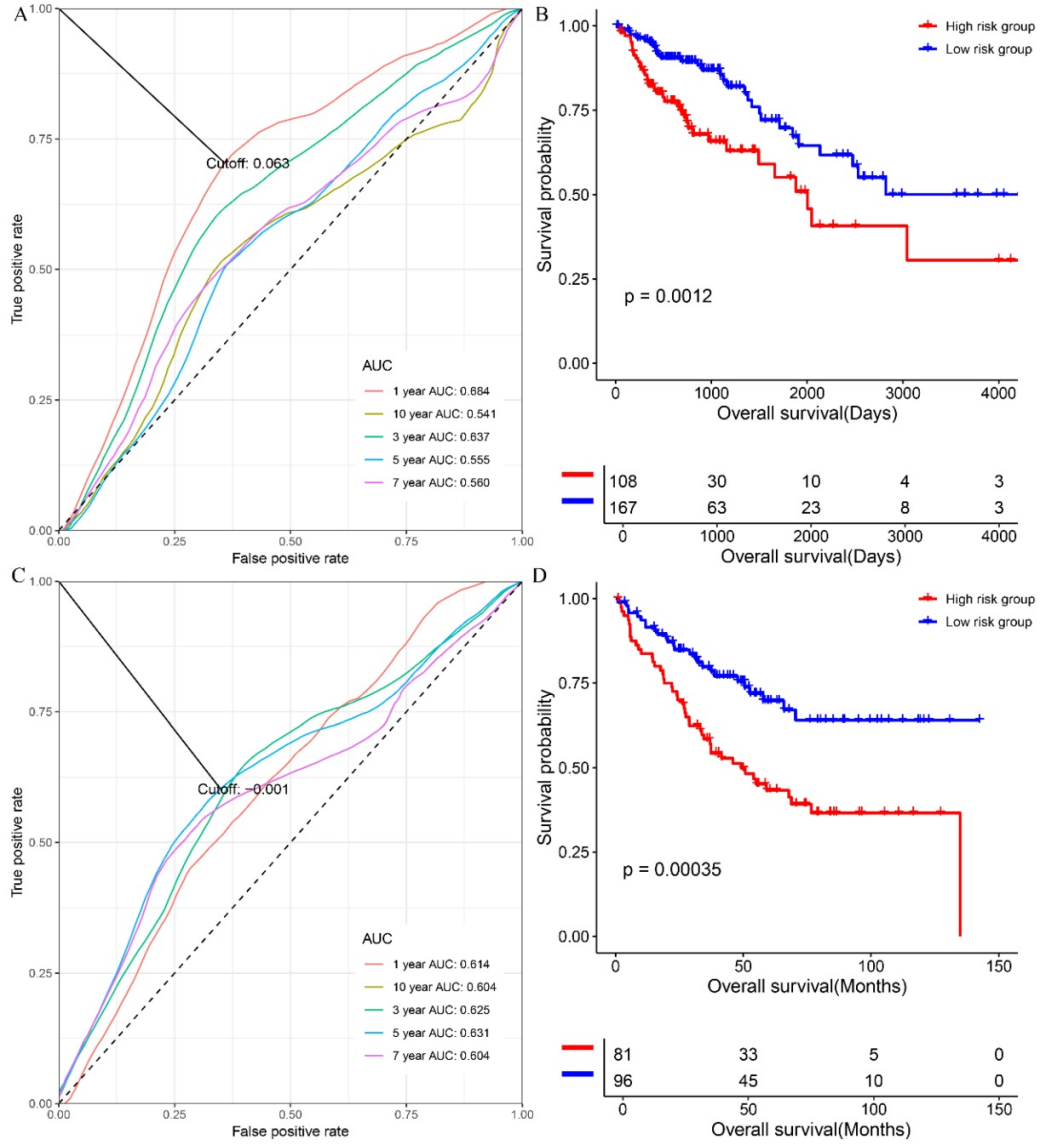
The performance of the multigene combination in predicting the overall survival patients with colon cancer in the TCGA-COAD and GSE17536. (A) Time-dependent analysis in the TCGA-COAD. (B) Overall survival differences of patients in the GSE17536. (C) Time-dependent analysis in the TCGA-COAD. (B) Overall survival differences of patients in the GSE17536.

At first, we investigated the performance of the multigene signature in predicting the OS of colon cancer patients. As shown in figure 1A, the time-dependent ROC curve suggested that the multigene panel showed a good performance in predicting OS of colon patients in the training set (The area under curves (AUCs) at one-year, three year, five-year, seven-year, ten-year, and fifteen-year were 0.714, 0.627, 0.649, 0.642, 0.651 and 0.669, respectively), and the multigene panel could classify the colon samples into different risk groups (HR=0.4928, 95% CI: 0.3341~0.727, log-rank P=0.00027, supplementary table s5 and figure 1B). Meanwhile, as shown in figure 1C, the multigene panel also show good prognostic performance at one-year (AUC: 0.634), three year (AUC:0.643), five-year (AUC: 0.623), seven-year (AUC: 0.619), ten-year (AUC: 0.628), and fifteen-year (AUC: 0.683), and the multigene signature could significantly classify patients into different risk groups in the test set(figure 1D, supplementary table s6). Meanwhile, we have validated the prognostic performance of the multigene panel in two independent validation cohort, and the results of time-dependent ROC analysis and KM curves suggested that the multigene panel could divide colon cancer patients into high-risk group and low-risk group in the TCGA-COAD (figure 2A, figure 2B, and supplementary table s7) and GSE17536 (figure 2C, figure 2D, and supplementary table s8).

Moreover, we also assessed the value of the multigene panel when predicting the RFS or DFS of colon cancer patients. The result of KM curves and CPH models suggested that patients in multigene panel low-risk group have better recurrence-free survival or disease-free survival compared with those in the multigene high-risk group in the training set (Log-rank P=0.039, supplementary figure s2A and supplementary table s9), test set (Log-rank P<0.0001, supplementary figure s2B and supplementary table s10), TCGA-COAD cohort (Log-rank P=0.0066, supplementary figure s2C and supplementary table s11) and GSE17536 cohort (Log-rank P<0.0001, supplementary figure 2D and supplementary table s12).

### Clinical application of the multigene panel

**Figure 3:**
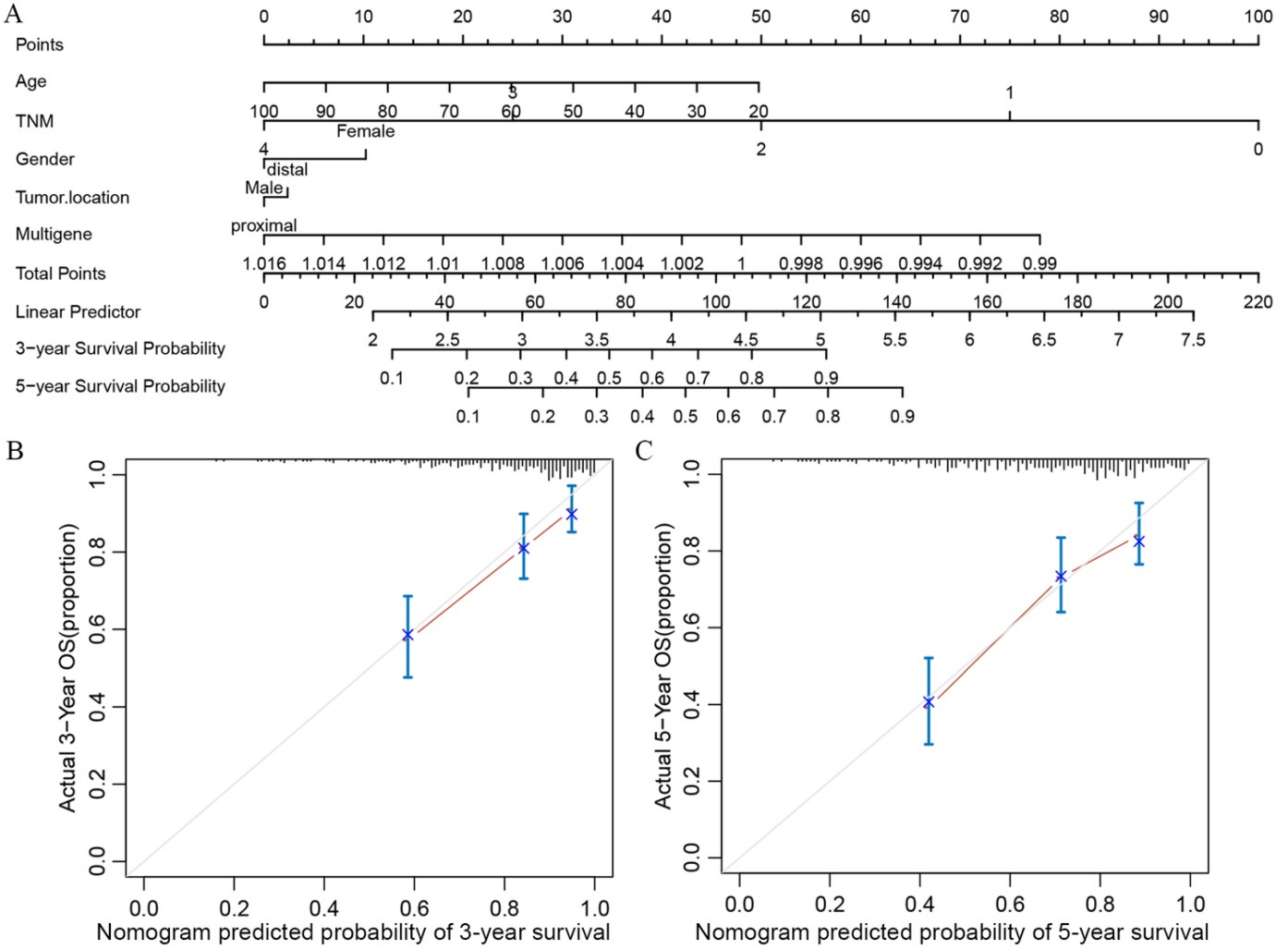
Nomogram and its associated calibration curve analysis. (A)Multigene based nomogram predicting the 3- and 5-year survival probability in patients with colon cancer. (B) Calibration analysis of the multigene containing nomogram at 3 years. (C) Calibration analysis of the multigene containing nomogram at 5 years.

**Figure 4:**
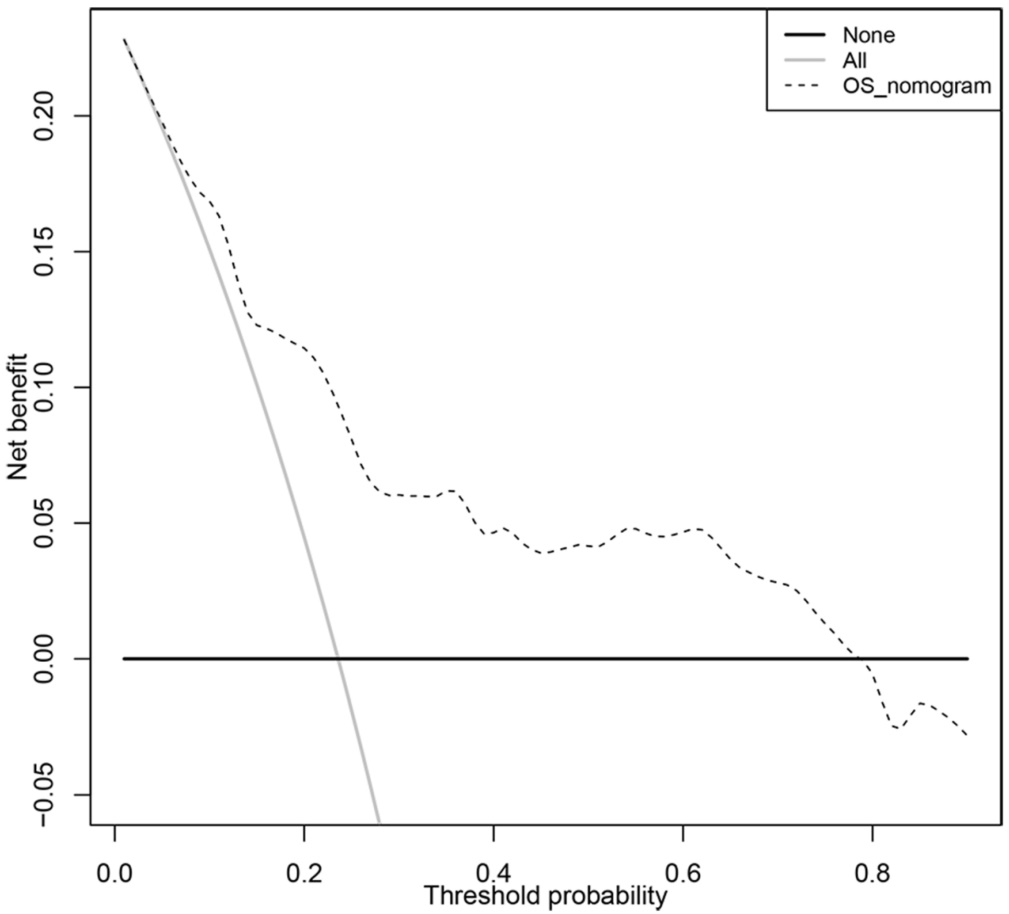
Decision curve analysis of the clinical use of the multigene based nomogram.

In order to transform our multigene panel into clinical application, we integrated patient age, gender, TNM stage, and tumor location, and the multigene panel to build a nomogram that predicted the 3-year survival probability and 5-year survival probability of colon cancer patients. In the nomogram, each variable corresponds to a score on the Points line, and the sum of the scores corresponding to all variables also has a score on the “Total points” line, then, then 3-year survival probability and 5-year probability of a patient can be estimated by his score on the “Total points” line (figure 3A). The calibration plot closely resembled the ideal diagonal curve at 3-year and 5-year (figure 3B and figure 3C). The C-indexes for internally validation and externally validation of the nomogram were 0.715 and 0.726, suggesting that the performance of the nomogram was reliable. Moreover, we have performed decision curve analysis (DCA) of the nomogram, as shown in figure 4, the multigene containing nomogram performed better at the threshold probability ranging from 3% to 77%.

### The prognostic performance of our multigene panel was comparable with existing biomarkers

As mentioned above, we compared the performance of our multigene panel with 10 existing biomarkers (including a 4-gene signature 23, a 15-gene signature 24, two 6-gene signatures^25^, ^28^, a 10-gene signature^26^, a 5-gene signature^27^, AEBP1^29^, FZD7^30^, CDX2^31^, MUC2^31^, PPM1H^32^, and LAYN^33^). As shown in figure 6, the C-index of our multigene panel was significantly higher or comparable with the existing biomarkers in the training set, test set, TCGA-COAD, and GSE17536, indicating that our multigene panel had comparable prognostication performance.

### Functional enrichment analysis using GSEA

**Figure 5:**
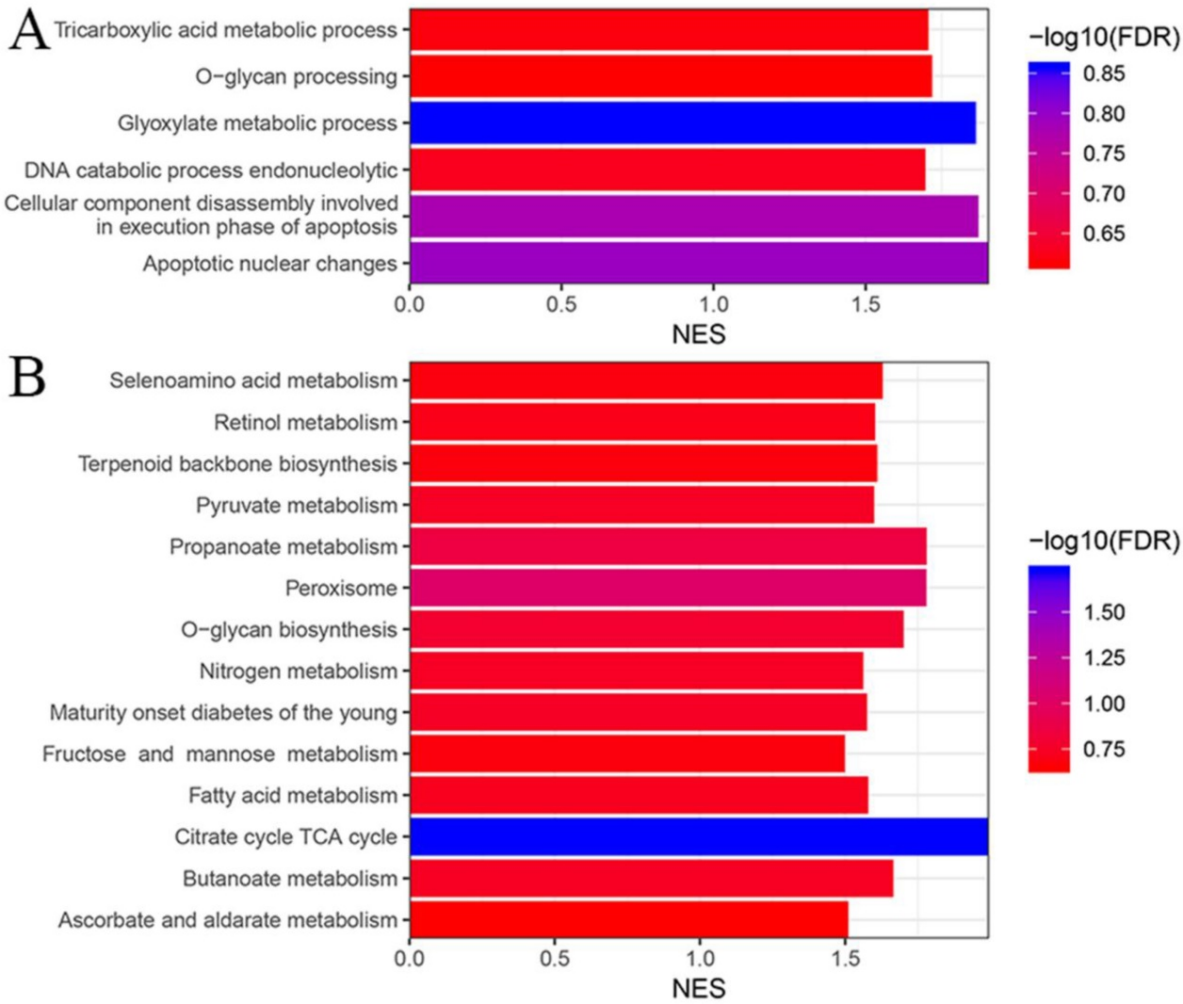
Gene ontology (A) and Kyoto Encyclopedia of Genes and Genomes (B) enrichment analysis based on the risk score of each colon cancer patients using gene set enrichment analysis.

**Figure 6:**
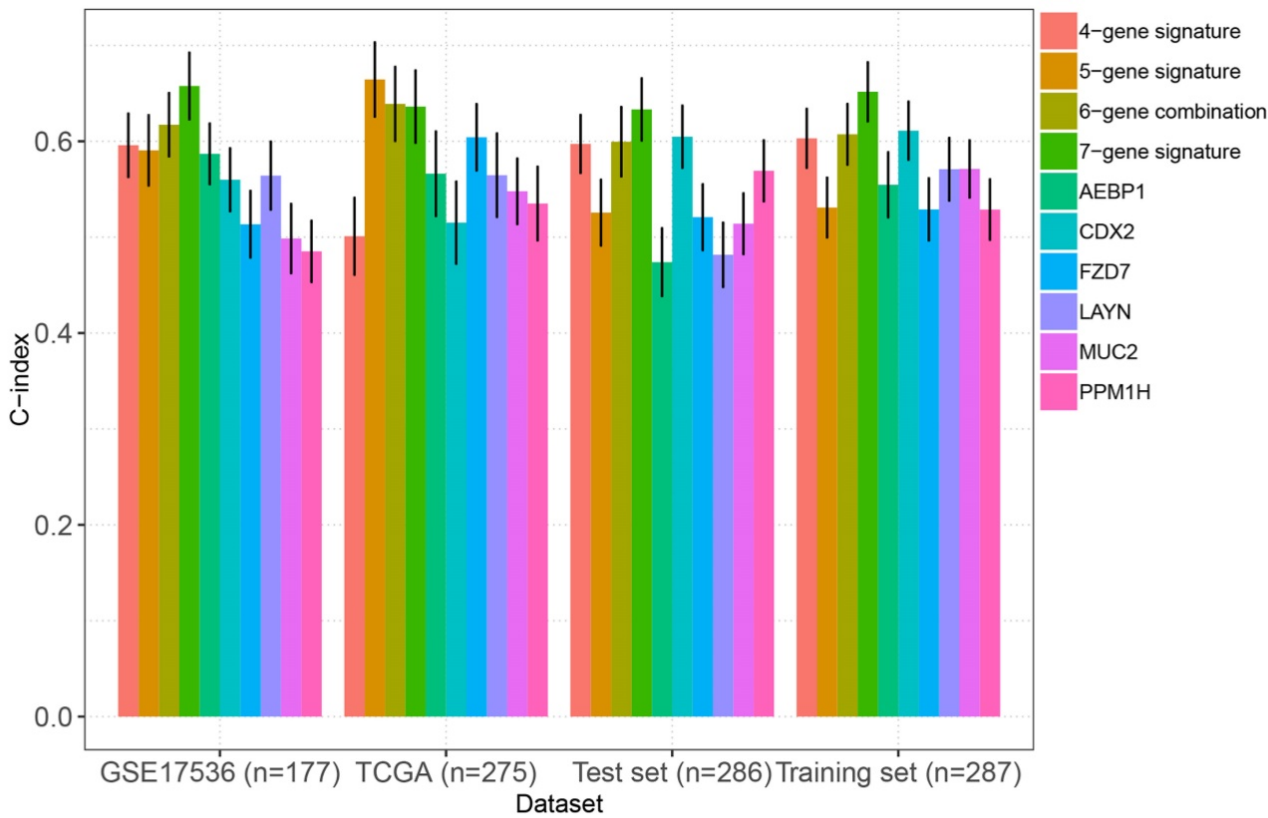
Comparison of the C-indexes between the multigene panel and other existing biomarkers in colon cancer

As mentioned in the method section, we performed GO and KEGG enrichment analysis to get a general knowledge of the functional role of the multigene panel using GSEA. As shown in figure 5A, colon samples in the multigene panel low risk group were significantly (P<0.05, FDR<25%) enriched in GO terms including glyoxylate metabolic process, apoptotic nuclear changes, cellular component disassembly involved in execution phase of apoptosis, DNA catabolic process endonucleolytic, tricarboxylic acid metabolic process, and O-glycan processing. Meanwhile, figure 5B indicated that samples in the multigene panel low risk group was significantly enriched in several KEGG pathways including citrate cycle TCA cycle, peroxisome, O-glycan biosynthesis, propanoate metabolism, butanoate metabolism, retinol metabolism, selenoamino acid metabolism, maturity onset diabetes of the young, nitrogen metabolism, pyruvate metabolism, terpenoid backbone biosynthesis, ascorbate and aldarate metabolism, fatty acid metabolism and fructose and mannose metabolism.

## Discussions

In the present study, we tried to develop a combination of multigene biomarkers (MYB, MSLN, INHBB, DCBLD2, MAP1B, PRELID2, and SH3RF2) by using ENCPH model. The assessment of the prognostication value of the multigene panel was performed on a total of 1,025 colon cancer patients (One training set, one internally validation set, and two externally validation cohorts), and the results of time-dependent ROC analysis and KM curves suggested that the multigene panel could stratified colon patients into notably different risk groups, and the results multivariable CPH model indicated that the multigene panel was an independent predictor for the OS and RFS/DFS of patients with colon cancer.

Actually, among the 7 genes included, several have been reported to be involved in the pathogenesis of colon cancer. Activation of MYB could induce colon tumorigenesis 35, and it was also selected as a target for antineoplastic therapy^36^. MSLN had been accepted to be a candidate biomarker in colon cancer 37. Qian Z et al. demonstrated that INHBB predicted worse survival rates in patients with colorectal cancer^38^ DCBLD2 was also identified as one of survival markers genes in colon cancer through consistent transcriptomic profiling by Martinez-Romero J et al.^26^. Gylfe AE et al. performed exome sequencing on a total of 25 colorectal cancer and corresponding healthy colon tissues, demonstrating that MAP1B was one of the candidate oncogene in patients with colon cancer 39. Kim TW et al. demonstrated that SH3RF2 was significantly increased in colon cancer cells, and higher expression of SH3RH2 was associated with progression, early relapse and poor survival 40. Thus, we have reasons to believe that the prognosis performance of the multigene panel was reliable.

As stated above, nomogram has been widely used in clinical settings, especially in the prediction and evaluation of survival of cancer patients with its easy-to-understand. Our nomogram integrated multigene, patient age, gender, TNM staging, and tumor location, which allowed clinicians to intuitively predict 3-year and 5-year survival rates of colon patients based on these clinical parameters. At the same time, internal and external verification results showed that both C-indexes for nomogram exceeded 0.7, which guaranteed the accuracy and reliability of nomogram prediction performance.

GSEA analysis based on GO and KEGG showed that low-risk colon cancer samples were mainly enriched in biological processes or pathways involved in cellular metabolism such as glyoxylate metabolic process, DNA catabolic process endonucleolytic, tricarboxylic acid metabolic process, etc. These results indicated that the seven genes included in the multigene panel might affect colon cancer through cellular metabolism.

This study included three independent colon cancer studies. However, owning to the clinical information of patients reported in different studies was inconsistent; the variables included multivariable analysis was not the same as each other. For example, in GSE39582, we included age, gender, tumor location, TNM staging, and multigene panel. However, in TCGA-COAD cohort, we included age, gender, tissue type, and preoperative CEA levels. This might cause the results to be biased to some extent. Therefore, cautions should be reserved when interpreting the prognosis roles of the multigene panel and the nomogram.

Taken together, the multigene panel we introduced showed considerable prognosis performance in colon cancer, and the multigene panel containing nomogram would help clinicians assess long-term survival probability.

## Supplementary Material

Supplementary figures and tables.
