# Supplementary material for: Multigene panel predicting survival of patients with colon cancer
Source: J Cancer. 2019 Nov 1;10(27):6792–800. doi: 10.7150/jca.35902 (PMC6909949; doi:10.7150/jca.35902)
Supplement: Supplementary file 1 — Supplementary figures and tables. [file jcav10p6792s1.pdf]

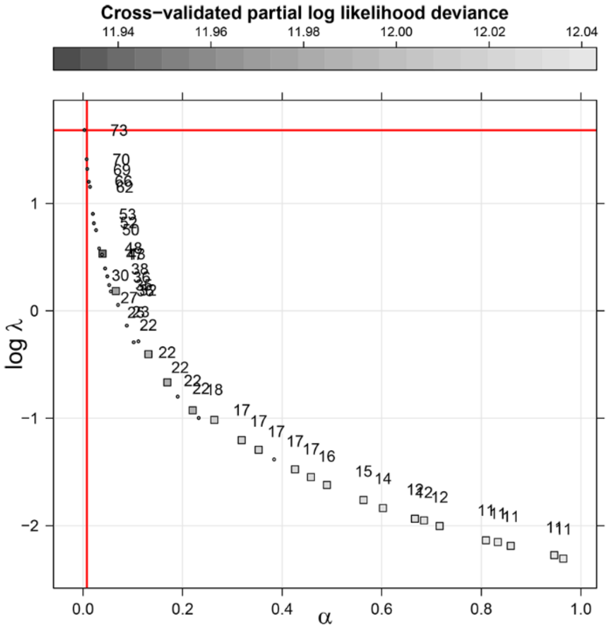


**Supplementary figure 1** Hyperparameters α and λ tuning in the elastic net using 10-fold cross-validation.

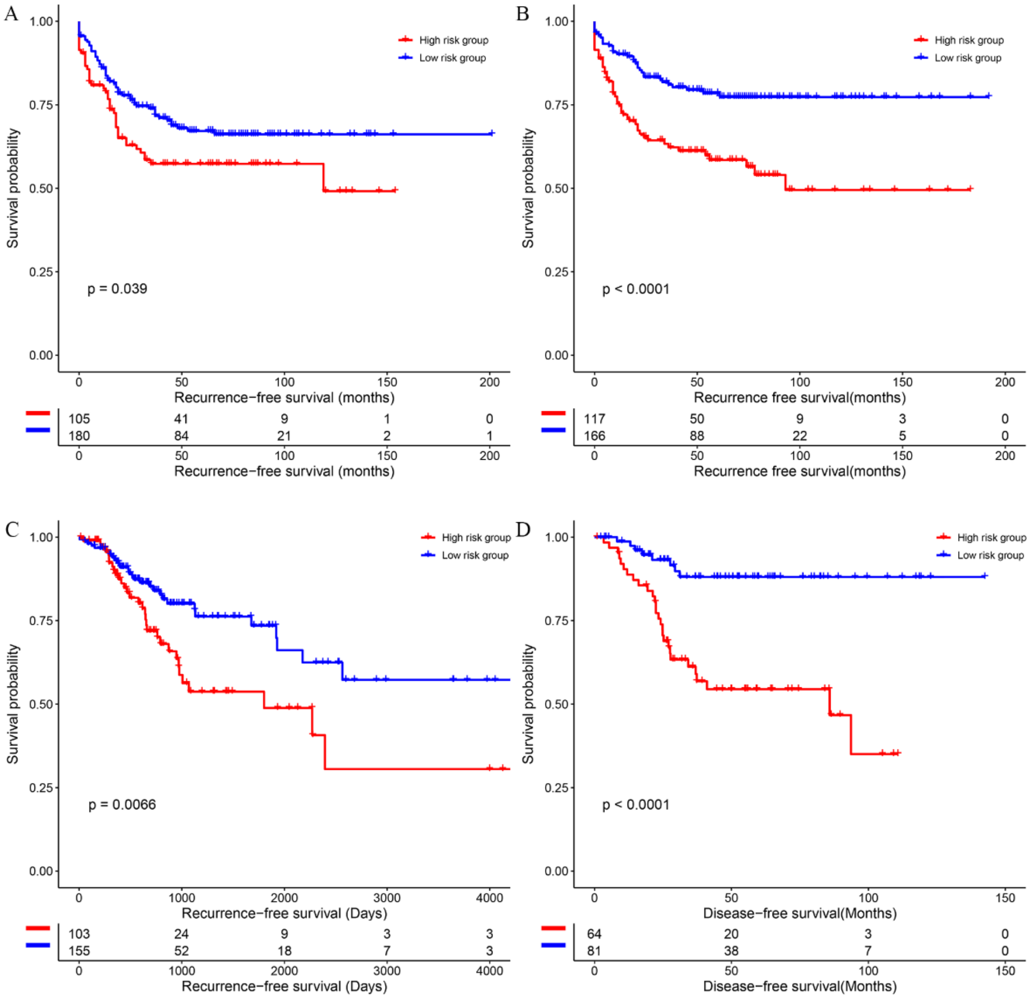


**Supplementary figure 2** Differences in recurrence/disease free survival of patients in the training set(A), test set (B), TCGA-COAD (C) and (D) GSE17536

**Supplementary table 1** Demographic characteristics of patients with colon cancer in the training set and test set

| Variable | Training set | | Test set | |
|---|---|---|---|---|
| | High risk group | Low risk group | High risk group | Low risk group |
| Gender | | | | |
| Male | 47 | 106 | 47 | 94 |
| Female | 58 | 76 | 70 | 75 |
| Age, median~range, years | 68(31-93) | 67(22-97) | 71.65(24.9-96) | 68(30.2-96) |
| T stage | | | | |
| T0 | 0 | 0 | 0 | 1 |
| T1 | 1 | 5 | 2 | 4 |
| T2 | 3 | 20 | 4 | 21 |
| T3 | 67 | 116 | 80 | 109 |
| T4 | 28 | 36 | 25 | 28 |
| Tis | 0 | 1 | 0 | 2 |
| NA | 6 | 4 | 6 | 4 |
| N stage | | | | |
| N0 | 53 | 104 | 58 | 92 |
| N1 | 21 | 43 | 26 | 44 |
| N2 | 22 | 28 | 25 | 25 |
| N3 | 2 | 1 | 1 | 2 |
| NA | 7 | 6 | 7 | 6 |
| M stage | | | | |
| M0 | 80 | 160 | 96 | 155 |
| M1 | 17 | 18 | 15 | 10 |
| NA | 8 | 4 | 6 | 4 |
| Tumor location | | | | |
| Distal | 57 | 117 | 76 | 96 |
| Proximal | 48 | 65 | 41 | 73 |
| overall survival, months | 46 (2-154) | 54.5(1-201) | 50(1-183) | 57(1-192) |
| Recurrence-free survival, months | 33(0-154) | 45(0-201) | 37(0-183) | 51.5(0-192 |

**Supplementary table 2** Demographic characteristic of patients with colon cancer in the TCGA-COAD cohort

| Variable | High risk group | Low risk group |
|---|---|---|
| Age, median~ range, years | 66.5(34-90) | 67(31-90) |
| Gender | | |
| Male | 62 | 89 |
| Female | 46 | 78 |
| Histological type | | |

| | | |
|---|---|---|
| Colon adenocarcinoma | 87 | 149 |
| Colon mucinous adenocarcinoma | 20 | 16 |
| NA | 1 | 2 |
| Pathological stage | | |
| I | 9 | 34 |
| II | 28 | 71 |
| III | 35 | 33 |
| IV | 22 | 9 |
| NA | 14 | 20 |
| Preoperative CEA, median~range | 4.2(0-1286) | 2.8(0.2-286.6) |
| Overall survival, days | 630(16-4502) | 701(14-4270) |
| Recurrence-free survival, days | 579(16-4502) | 666(6-4270) |

**Supplementary table 3** Demographic characteristics of patients with colon cancer in the GSE17536 cohort

| Variable | High risk group | Low risk group |
|---|---|---|
| Age, median~range, years | 65(39-92) | 67(26-89) |
| Gender | | |
| Male | 46 | 46 |
| Female | 35 | 50 |
| Race | | |
| Black | 6 | 3 |
| Caucasian | 66 | 85 |
| Hispanic | 1 | 0 |
| Other | 8 | 8 |
| AJCC stage | | |
| 1 | 4 | 20 |
| 2 | 25 | 32 |
| 3 | 33 | 24 |
| 4 | 19 | 20 |
| Grade | | |
| 1 | 5 | 11 |
| 2 | 59 | 75 |
| 3 | 17 | 10 |
| Overall survival, months | 37.31(0.92-134.86) | 48.48(0.92-142.55) |
| Disease-free survival | 37.31(0.92-134.86) | 48.48(0.92-142.55) |

**Supplementary table 4** Overall survival associated genes identified with elastic net

| **Gene symbol** | **Gene name** | **Coefficient** |
|---|---|---|
| MYB | MYB proto-oncogene, transcription factor | -0.00045 |
| MSLN | mesothelin | 0.001898 |
| INHBB | inhibin subunit beta B | 0.00161 |
| DCBLD2 | discoidin, CUB and LCCL domain containing 2 | 0.003451 |
| MAP1B | microtubule-associated protein 1B | 0.003582 |

| | | |
|---|---|---|
| PRELID2 | PRELID2 | -0.00064 |
| SH3RF2 | SH3 domain containing ring finger 2 | -0.00151 |

**Supplementary table 5** Cox proportional hazards regression model on the overall survival of colon patients in the training set

| Variable | Univariate analysis | | Multivariable analysis | |
|---|---|---|---|---|
| | HR (95% CI) | P value | HR (95% CI) | P value |
| 7-gene signature | 0.4928(0.3341~0.727)<br>Reference: 7-gene signature high risk | 0.0004 | 0.4796(0.3221~0.7139) | 0.0003 |
| Gender | 1.2045(0.8129~1.7846)<br>Reference: Female | 0.3537 | 1.5373(1.0196~2.3177) | 0.0401 |
| Age | 1.0143(0.999~1.0298) | 0.068 | 1.0228(1.007~1.0388) | 0.0045 |
| Tumor location | 1.0898(0.7321~1.6224)<br>Reference: Distal | 0.6717 | 0.9992(0.6619~1.5082) | 0.9969 |
| TNM | 2.4527(1.8684~3.2196) | <0.001 | 2.624(1.9704~3.4944) | <0.001 |

**Abbreviations:** HR, hazards ratio; CI, confidence interval;

**Supplementary table 6** Cox proportional hazards regression model on the overall survival of colon patients in the test set

| Variable | Univariate analysis | | Multivariable analysis | |
|---|---|---|---|---|
| | HR (95% CI) | P value | HR (95% CI) | P value |
| 7-gene signature | 0.4961(0.3256~0.7558)<br>Reference: 7-gene signature high risk | 0.0011 | 0.6647(0.444~0.995) | 0.0472 |
| Gender | 1.4802(0.9573~2.2887)<br>Reference: Female | 0.0778 | 1.3396(0.8907~2.0147) | 0.1603 |
| Age | 1.0394(1.0194~1.0597) | 0.0001 | 0.9998(0.9854~1.0144) | 0.9758 |
| Tumor location | 1.0972(0.714~1.6861)<br>Reference: Distal | 0.6723 | 0.7156(0.469~1.0919) | 0.1207 |
| TNM | 1.5156(1.1271~2.0379) | 0.0059 | 2.9945(2.2591~3.9692) | <0.001 |

**Abbreviations:** HR, hazards ratio; CI: confidence interval; TNM, tumor-node-metastasis stage

**Supplementary table 7** Cox proportional hazards regression model on the overall survival of colon patients in the TCGA colon cancer cohort

| Variable | Univariate analysis | | Multivariable analysis | |
|---|---|---|---|---|
| | HR (95% CI) | P value | HR (95% CI) | P value |
| 7-gene signature | 0.4627(0.2868~0.7467)<br>Reference: 7-gene signature high risk | 0.0016 | 0.4951(0.2123~1.1544) | 0.1036 |
| Age | 1.016(0.997~1.0354) | 0.1001 | 1.048(1.0121~1.0852) | 0.0084 |
| Gender | 1.4579(0.8919~2.3829)<br>Reference: Female | 0.1327 | 1.0803(0.4783~2.4399) | 0.8526 |

| | | | | |
|---|---|---|---|---|
| Histological type | 1.2561(0.64~2.4653) | 0.5075 | 1.5937(0.4422~5.7433) | 0.4762 |
| | Reference: Colon Adenocarcinoma | | | |
| Preoperative CEA | 1.0018(0.9996~1.0041) | 0.1101 | 0.9999(0.9971~1.0026) | 0.9214 |
| Stage | 1.2368(1.1299~1.354) | <0.001 | 1.4263(1.1942~1.7034) | 0.0001 |

**Abbreviations:** HR, hazards ratio; CI: confidence interval.

**Supplementary table 8** Cox proportional hazards regression model on the overall survival of colon patients in GSE17536

| Variable | Univariate analysis | | Multivariable analysis | |
|---|---|---|---|---|
| | **HR (95% CI)** | **P value** | **HR (95% CI)** | **P value** |
| 7-gene signature | 0.4281(0.2651~0.6913) | 0.0005 | 0.437(0.2596~0.7355) | 0.0018 |
| | Reference: 7-gene signature high risk | | | |
| Age | 1.0063(0.9884~1.0245) | 0.4919 | 1.018(0.9959~1.0406) | 0.1119 |
| Gender | 1.1049(0.694~1.7591) | 0.6741 | 0.828(0.4861~1.4105) | 0.4875 |
| | Reference: Female | | | |
| Race Caucasian | 0.4373(0.1885~1.0146) | 0.0541 | 0.5113(0.2025~1.2909) | 0.1557 |
| Race Hispanic | 0.3483(0.0337~3.5961) | 0.3759 | 0.4417(0.0169~11.5292) | 0.6234 |
| Race other | 0.2189(0.0615~0.7795) | 0.0191 | 0.2499(0.0662~0.943) | 0.0407 |
| | Reference: Black | | | |
| AJCC stage | 2.8548(2.1116~3.8595) | <0.001 | 3.151(2.2417~4.4291) | <0.001 |
| Grade | 2.0043(1.2493~3.2157) | 0.0039 | 1.2501(0.7773~2.0104) | 0.3573 |

**Abbreviations:** HR, hazards ratio; CI, confidence interval

**Supplementary table 9** Cox proportional hazards regression model on the recurrence-free survival of colon patients in the training set

| Variable | Univariate analysis | | Multivariable analysis | |
|---|---|---|---|---|
| | HR (95% CI) | P value | HR (95% CI) | P value |
| 7-gene signature | 0.6568(0.4406~0.9791) | 0.039 | 0.6647(0.444~0.995) | 0.0472 |
| | Reference: 7-gene signature high risk | | | |
| Gender | 1.1607(0.7788~1.7298) | 0.4642 | 1.3396(0.8907~2.0147) | 0.1603 |
| | Reference: Female | | | |
| Age | 0.9964(0.9822~1.0108) | 0.6247 | 0.9998(0.9854~1.0144) | 0.9758 |
| Tumor location | 0.7929(0.5229~1.2021) | 0.2744 | 0.7156(0.469~1.0919) | 0.1207 |
| | Reference: Distal | | | |
| TNM | 2.8982(2.2039~3.8112) | <0.001 | 2.9945(2.2591~3.9692) | <0.001 |

**Abbreviations:** HR, hazards ratio; CI: confidence interval; TNM, tumor-node-metastasis stage

**Supplementary table 10** Cox proportional hazards regression model on the recurrence-free survival of colon patients in the test set

| Variable | Univariate analysis | | Multivariable analysis | |
|---|---|---|---|---|
| | HR (95% CI) | P value | HR (95% CI) | P value |

| | | | | |
|---|---|---|---|---|
| 7-gene signature | 0.4198(0.2694~0.6542) | 0.0001 | 0.4534(0.2874~0.7154) | 0.0007 |
| | Reference: 7-gene signature high risk | | | |
| Gender | 1.5008(0.9484~2.3749) | 0.0829 | 1.6712(1.0522~2.6544) | 0.0296 |
| | Reference: Female | | | |
| Age | 1.0046(0.9868~1.0226) | 0.6181 | 1.0084(0.9893~1.0279) | 0.3926 |
| Tumor location | 0.7467(0.4704~1.1854) | 0.2155 | 0.7476(0.4638~1.2053) | 0.2326 |
| | Reference: Distal | | | |
| TNM | 2.3882(1.7205~3.315) | <0.001 | 2.4782(1.7766~3.4568) | <0.001 |

**Abbreviations:** HR, hazards ratio; CI: confidence interval; TNM, tumor-node-metastasis stage

**Supplementary table 11** Cox proportional hazards regression model on the recurrence-free survival of colon patients in the TCGA colon cancer cohort

| Variable | Univariate analysis | | Multivariable analysis | |
|---|---|---|---|---|
| | HR (95% CI) | P value | HR (95% CI) | P value |
| 7-gene signature | 0.5063(0.3071~0.8349) | 0.0077 | 0.5557(0.2713~1.1381) | 0.1082 |
| | Reference: 7-gene signature high risk | | | |
| Age | 0.9867(0.9678~1.0059) | 0.1742 | 0.9909(0.9626~1.02) | 0.5354 |
| Gender | 1.6052(0.9548~2.6988) | 0.0742 | 1.1677(0.5453~2.5005) | 0.6898 |
| | Reference: Female | | | |
| Histological type | 0.804(0.3455~1.8706) | 0.6126 | 1.7226(0.5879~5.0473) | 0.3215 |
| | Reference: Colon Adenocarcinoma | | | |
| Preoperative CEA | 1.0015(0.9964~1.0066) | 0.5717 | 0.9997(0.9901~1.0094) | 0.9479 |
| Pathological stage | 1.2005(1.0933~1.3182) | 0.0001 | 1.1902(1.0232~1.3846) | 0.024 |

**Abbreviations:** HR, hazards ratio; CI, confidence interval;

**Supplementary table 12** Cox proportional hazards regression model on the Disease-free survival of colon patients in the GSE17536

| Variable | **Univariate analysis** | | **Multivariable analysis** | |
|---|---|---|---|---|
| | HR (95% CI) | P value | HR (95% CI) | P value |
| 7-gene signature | 0.2003(0.0912~0.4401) | 0.0001 | 0.2014(0.089~0.4558) | 0.0001 |
| | Reference: 7-gene signature high risk | | | |
| Age | 0.9836(0.9611~1.0065) | 0.1597 | 0.9849(0.9586~1.0119) | 0.2709 |
| Gender | 0.9998(0.5195~1.9243) | 0.9996 | 0.6835(0.3223~1.4496) | 0.3212 |
| | Reference: Female | | | |
| Race Caucasian | 0.7255(0.1728~3.0456) | 0.6611 | 1.2422(0.2678~5.7617) | 0.7818 |
| Race Hispanic | 1.6926(0.1505~19.029) | 0.6699 | 1.9244(0.1486~24.9141) | 0.6164 |
| Race Other | 0.8153(0.1488~4.468) | 0.814 | 1.1849(0.1974~7.1133) | 0.8528 |
| | Reference: Black | | | |
| AJCC stage | 2.0465(1.3512~3.0996) | 0.0007 | 2.2459(1.3137~3.8396) | 0.0031 |

| | | | | |
|---|---|---|---|---|
| Grade | 1.8477(0.9376~3.6409) | 0.0761 | 1.2711(0.6274~2.5754) | 0.5055 |

**Abbreviations:** HR, hazards ratio; CI, confidence interval;
